# Poly[di-μ_2_-chlorido-tri-μ_2_-terephthalato-tetra­lead(II)]

**DOI:** 10.1107/S160053681101779X

**Published:** 2011-05-20

**Authors:** Lei Yang, Zhongyue Li, Guanghua Li

**Affiliations:** a The Department of Physics–Chemistry, Henan Polytechnic University, Jiao Zuo 454150, People’s Republic of China; b State Key Laboratory of Inorganic Synthesis and Preparative Chemistry, College of Chemistry, Jilin University, Changchun 130012, People’s Republic of China

## Abstract

The title compound, [Pb_4_(C_8_H_4_O_4_)_3_Cl_2_]_*n*_, consists of a three-dimensional inorganic–organic hybrid framework. The asymmetric unit contains two Pb^2+^ cations, one Cl^−^ anion and one and a half terephthalate anions, the latter being completed by inversion symmetry. The two Pb^2+^ cations are each surrounded by five O atoms and one Cl atom in the form of irregular polyhedra. The cations are linked by μ_2_-O and μ_2_-Cl atoms into binuclear units, which are further extended through Pb—O inter­actions into an undulated inorganic layer parallel to (001). These layers are connected along [001] by the terephthalate groups into a three-dimensional framework.

## Related literature

For a description of inorganic–organic hybrid compounds, see: Cheetham *et al.* (2006 ▶). For Pb—Cl bond lengths, see: Casas (2003 ▶). For a related structure, see: Zhang *et al.* (2009 ▶).
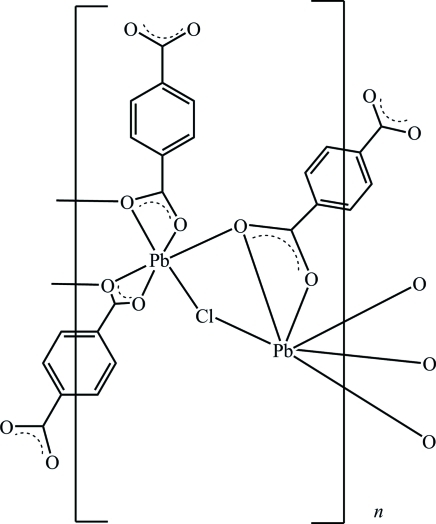


## Experimental

### 

#### Crystal data

[Pb_4_(C_8_H_4_O_4_)_3_Cl_2_]
*M* *_r_* = 1392.00
Monoclinic, 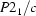

*a* = 5.9900 (1) Å
*b* = 11.8529 (2) Å
*c* = 18.4737 (3) Å
β = 91.778 (1)°
*V* = 1310.98 (4) Å^3^
*Z* = 2
Mo *K*α radiation
μ = 25.88 mm^−1^
*T* = 296 K
0.32 × 0.26 × 0.21 mm

#### Data collection

Bruker APEXII CCD diffractometer
Absorption correction: multi-scan (*SADABS*; Bruker, 2008 ▶) *T*_min_ = 0.000, *T*_max_ = 0.004
9476 measured reflections
2298 independent reflections
2097 reflections with *I* > 2σ(*I*)
*R*_int_ = 0.044

#### Refinement

*R*[*F* ^2^ > 2σ(*F* ^2^)] = 0.032
*wR*(*F* ^2^) = 0.092
*S* = 1.00
2298 reflections
192 parameters
H-atom parameters constrained
Δρ_max_ = 2.29 e Å^−3^
Δρ_min_ = −1.89 e Å^−3^

### 

Data collection: *APEX2* (Bruker, 2008 ▶); cell refinement: *SAINT* (Bruker, 2008 ▶); data reduction: *SAINT*; program(s) used to solve structure: *SHELXTL* (Sheldrick, 2008 ▶); program(s) used to refine structure: *SHELXTL*; molecular graphics: *ORTEP-3* (Farrugia, 1997 ▶); software used to prepare material for publication: *SHELXTL*.

## Supplementary Material

Crystal structure: contains datablocks I, global. DOI: 10.1107/S160053681101779X/wm2483sup1.cif

Structure factors: contains datablocks I. DOI: 10.1107/S160053681101779X/wm2483Isup2.hkl

Supplementary material file. DOI: 10.1107/S160053681101779X/wm2483Isup3.cdx

Additional supplementary materials:  crystallographic information; 3D view; checkCIF report

## Figures and Tables

**Table 1:** Selected bond lengths (Å)

Pb1—O2	2.407 (7)
Pb1—O1	2.572 (6)
Pb1—O6^i^	2.586 (6)
Pb1—O3	2.618 (6)
Pb1—O4^ii^	2.743 (6)
Pb1—Cl1	2.893 (2)
Pb2—O5^i^	2.408 (6)
Pb2—O4	2.516 (6)
Pb2—O3	2.616 (6)
Pb2—O6^i^	2.696 (7)
Pb2—O1^iii^	2.712 (6)
Pb2—Cl1^iii^	3.010 (2)

Symmetry codes: (i) 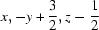; (ii) 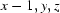; (iii) 
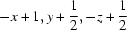
.

**Fig. 1.**
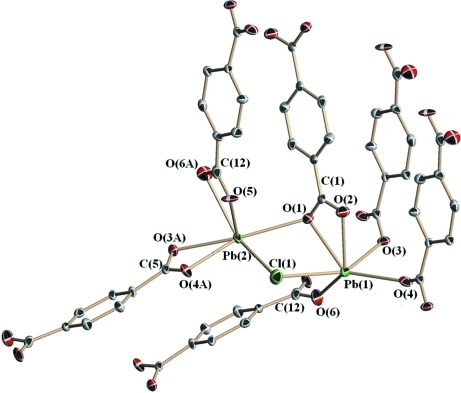
An ORTEP plot of the Pb-environment in the title compound showing 50% probability ellipsoids and the atom labeling scheme.

**Fig. 2.**
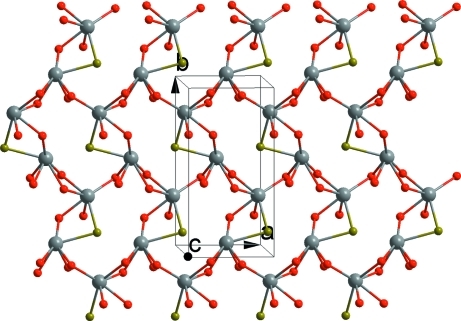
The (001) plane of the title compound, dispaying a wave-like 2-D 12-ring inorganic network with Pb atoms (grey) linked by O (red) and Cl atoms (green).

**Fig. 3.**
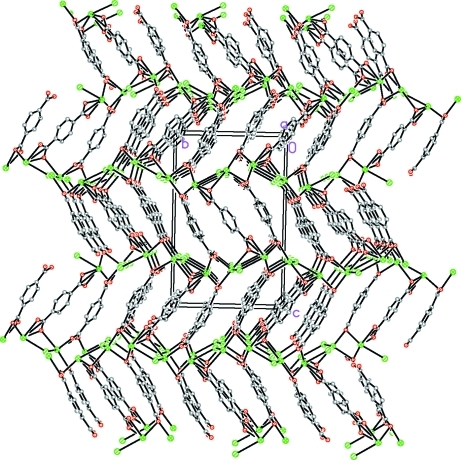
View of the structure along [100], showing the connection of the inorganic layers by terephthalic groups along [001].

## supplementary crystallographic information

### Comment

According to the classification scheme of Cheetham *et al.* (2006), hybrid compounds are divided into two categories: metal-organic coordination polymers and extended inorganic hybrids. In contrast to metal-organic coordination polymers, extended inorganic hybrids which contain extended arrays of inorganic connectivity, *M*—*X*—*M* (*M* = metal, *X* = O, Cl, N, and S), have been scarcely investigated. Up to now, only some lead-containing compounds with aromatic carboxylate ligands forming an extended inorganic hybrid network have been reported (Zhang *et al.*, 2009).

The asymmetric unit of the title compound, [Pb_4_(C_8_H_4_O_4_)_3_Cl_2_]_n_, consists of two Pb^2+^ cations, one Cl^-^ anion and one and a half crystallographically independent terephthalate ligands. The C5 containing terephthalate ligand lies in a general position and the other half one is located at a center of inversion. An *ORTEP* drawing of the coordination environment of the Pb^2+^ cations is shown in Figure 1. The two unique lead atoms are both six-coordinate by five O atoms from tetephtalate anions and one Cl atom in the form of irregular polyhedra. Pb1 is connected to O1 and O2 from a bidentately coordinating anion, to O3, O4 and O6 from three monodentately coordinating anions and to one Cl atom. Pb2 is coordinated by O3, O4, O5, O6 from two bidentatly coordinating anions and by O1 from a monodentately coordinating anion and by one Cl atom. Pb1 and Pb2 atoms are linked by *µ*_2_-O1 and *µ*_2_-Cl1 to form a binuclear unit. These units are further extended into a wave-like 2-D inorganic layer parallel to (001) by sharing additional O atoms (Fig. 2). The Pb—O bond lengths are in the range of 2.4077 (7) to 2.743 (6) Å. The Pb—Cl bond lengths lie in the range of 2.893 (2) to 3.010 (2) Å, values comparable with similar structures (Casas, 2003). The parallel 2-D inorganic layers are connected along [001] by terephthalic groups to form the 3-D framework, as shown in Figure 3.

It is worth noticing that it is rare that in the inorganic layers adjacent Pb atoms are linked by one bridging chloride atom and one bridging oxygen atom other than two Cl or O atoms.

### Experimental

The title compound was synthesized under hydrothermal conditions. All reagents were of analytical grade. A suspension of Pb(NO_3_)_2_ (1 mmol) and 4,4'-bipyridine (2 mmol) in water (5 ml) was slowly added into a solution of terephthalic acid (2 mmol) in ethanol (10 ml) and was subsequently stirred. The resulting mixture was further stirred for 4 h at 393 K, and then filtered off. The filtrate pH was adjusted to 3 by hydrochloric acid. The final reaction mixture was heated in a sealed Teflon-lined steel autoclave at 453 K for 7 days. The autoclave was then cooled down to room temperature and colourless block-shaped single crystals were isolated, washed with distilled water and dried in air.

### Refinement

H atoms were positioned geometrically, with C—H = 0.93 Å and U_iso_(H) = 1.2U_eq_(C). The deepest hole and the highest remaining peak in the difference map are located 1.00 Å from Pb1 and 0.88 Å from Pb2, respectively.

### Crystal data

**Table:**

[Pb_4_(C_8_H_4_O_4_)_3_Cl_2_]	*F*(000) = 1228
*M**_r_* = 1392.00	*D*_x_ = 3.526 Mg m^−^^3^
Monoclinic, *P*2_1_/*c*	Mo *K*α radiation, λ = 0.71073 Å
Hall symbol: -P 2ybc	Cell parameters from 946 reflections
*a* = 5.9900 (1) Å	θ = 2.6–25.0°
*b* = 11.8529 (2) Å	µ = 25.88 mm^−^^1^
*c* = 18.4737 (3) Å	*T* = 296 K
β = 91.778 (1)°	Block, colourless
*V* = 1310.98 (4) Å^3^	0.32 × 0.26 × 0.21 mm
*Z* = 2	

### Data collection

**Table:**

Bruker APEXII CCD diffractometer	2298 independent reflections
Radiation source: fine-focus sealed tube	2097 reflections with *I* > 2σ(*I*)
graphite	*R*_int_ = 0.044
ω scans	θ_max_ = 25.0°, θ_min_ = 2.0°
Absorption correction: multi-scan (*SADABS*; Bruker, 2008)	*h* = −7→7
*T*_min_ = 0.000, *T*_max_ = 0.004	*k* = −14→12
9476 measured reflections	*l* = −21→21

### Refinement

**Table:**

Refinement on *F*^2^	Secondary atom site location: difference Fourier map
Least-squares matrix: full	Hydrogen site location: inferred from neighbouring sites
*R*[*F*^2^ > 2σ(*F*^2^)] = 0.032	H-atom parameters constrained
*wR*(*F*^2^) = 0.092	*w* = 1/[σ^2^(*F*_o_^2^) + (0.0714*P*)^2^] where *P* = (*F*_o_^2^ + 2*F*_c_^2^)/3
*S* = 1.00	(Δ/σ)_max_ = 0.001
2298 reflections	Δρ_max_ = 2.29 e Å^−^^3^
192 parameters	Δρ_min_ = −1.89 e Å^−^^3^
0 restraints	Extinction correction: *SHELXTL* (Sheldrick, 2008), Fc^*^=kFc[1+0.001xFc^2^λ^3^/sin(2θ)]^-1/4^
Primary atom site location: structure-invariant direct methods	Extinction coefficient: 0.0050 (3)

### Special details

**Table:**

Geometry. All e.s.d.'s (except the e.s.d. in the dihedral angle between two l.s. planes) are estimated using the full covariance matrix. The cell e.s.d.'s are taken into account individually in the estimation of e.s.d.'s in distances, angles and torsion angles; correlations between e.s.d.'s in cell parameters are only used when they are defined by crystal symmetry. An approximate (isotropic) treatment of cell e.s.d.'s is used for estimating e.s.d.'s involving l.s. planes.
Refinement. Refinement of *F*^2^ against ALL reflections. The weighted *R*-factor *wR* and goodness of fit *S* are based on *F*^2^, conventional *R*-factors *R* are based on *F*, with *F* set to zero for negative *F*^2^. The threshold expression of *F*^2^ > σ(*F*^2^) is used only for calculating *R*-factors(gt) *etc*. and is not relevant to the choice of reflections for refinement. *R*-factors based on *F*^2^ are statistically about twice as large as those based on *F*, and *R*- factors based on ALL data will be even larger.

### Fractional atomic coordinates and isotropic or equivalent isotropic displacement parameters (Å^2^)

**Table:**

	*x*	*y*	*z*	*U*_iso_*/*U*_eq_	
Pb1	0.06200 (5)	0.80676 (3)	0.303842 (18)	0.01898 (17)	
Pb2	0.55452 (5)	1.03175 (2)	0.274731 (18)	0.01933 (17)	
O3	0.3541 (9)	0.9266 (5)	0.3789 (3)	0.0218 (13)	
O1	0.3892 (9)	0.6746 (5)	0.3379 (4)	0.0225 (13)	
O2	0.0928 (10)	0.6826 (5)	0.4068 (4)	0.0294 (15)	
O4	0.7213 (10)	0.9102 (5)	0.3742 (3)	0.0267 (14)	
C9	0.5436 (13)	0.7355 (7)	0.6138 (5)	0.0172 (17)	
C6	0.5453 (14)	0.8489 (6)	0.4809 (5)	0.0187 (18)	
C8	0.3524 (13)	0.7937 (6)	0.5872 (5)	0.0185 (18)	
H8	0.2240	0.7939	0.6142	0.022*	
C7	0.3522 (13)	0.8504 (7)	0.5216 (5)	0.0190 (18)	
H7	0.2256	0.8890	0.5049	0.023*	
C10	0.7317 (13)	0.7385 (7)	0.5731 (5)	0.0210 (19)	
H10	0.8601	0.7021	0.5904	0.025*	
C5	0.5390 (13)	0.8979 (6)	0.4063 (4)	0.0163 (17)	
C11	0.7360 (13)	0.7938 (7)	0.5077 (5)	0.0185 (18)	
H11	0.8660	0.7944	0.4814	0.022*	
O5	0.7114 (9)	0.6137 (5)	0.7009 (4)	0.0289 (15)	
O6	0.3527 (11)	0.6448 (6)	0.7080 (4)	0.0341 (16)	
C12	0.5354 (15)	0.6614 (7)	0.6791 (5)	0.024 (2)	
C3	0.2855 (14)	0.5387 (6)	0.5111 (5)	0.0192 (19)	
H3	0.1419	0.5653	0.5184	0.023*	
C1	0.2880 (13)	0.6461 (6)	0.3939 (5)	0.0175 (18)	
C4	0.6133 (14)	0.5318 (6)	0.4382 (5)	0.022 (2)	
H4	0.6893	0.5533	0.3973	0.026*	
C2	0.3971 (13)	0.5706 (6)	0.4485 (5)	0.0188 (18)	
Cl1	−0.0398 (3)	0.5962 (2)	0.23077 (13)	0.0308 (6)	

### Atomic displacement parameters (Å^2^)

**Table:**

	*U*^11^	*U*^22^	*U*^33^	*U*^12^	*U*^13^	*U*^23^
Pb1	0.0220 (2)	0.0166 (2)	0.0183 (3)	0.00318 (11)	0.00007 (15)	0.00127 (12)
Pb2	0.0310 (2)	0.0113 (2)	0.0157 (3)	0.00216 (11)	0.00038 (15)	0.00016 (12)
O3	0.026 (3)	0.014 (3)	0.026 (4)	0.002 (2)	−0.004 (3)	0.009 (3)
O1	0.026 (3)	0.017 (3)	0.024 (4)	0.003 (2)	0.000 (3)	0.003 (3)
O2	0.031 (3)	0.030 (4)	0.028 (4)	0.008 (3)	0.003 (3)	0.003 (3)
O4	0.033 (3)	0.032 (4)	0.015 (4)	0.003 (3)	0.004 (3)	0.005 (3)
C9	0.026 (4)	0.015 (4)	0.011 (5)	−0.005 (3)	−0.003 (3)	−0.003 (3)
C6	0.033 (4)	0.006 (4)	0.017 (5)	0.005 (3)	0.001 (4)	0.003 (3)
C8	0.022 (4)	0.013 (4)	0.021 (5)	−0.003 (3)	0.001 (4)	0.000 (3)
C7	0.027 (4)	0.011 (4)	0.020 (5)	−0.002 (3)	0.005 (4)	−0.005 (3)
C10	0.020 (4)	0.021 (5)	0.022 (5)	0.001 (3)	0.000 (4)	0.004 (4)
C5	0.029 (4)	0.008 (4)	0.012 (4)	−0.003 (3)	0.000 (3)	−0.002 (3)
C11	0.015 (4)	0.020 (4)	0.020 (5)	0.000 (3)	0.000 (3)	0.000 (4)
O5	0.031 (3)	0.026 (3)	0.029 (4)	0.001 (3)	−0.005 (3)	0.017 (3)
O6	0.038 (3)	0.034 (4)	0.031 (4)	−0.002 (3)	0.011 (3)	0.008 (3)
C12	0.039 (5)	0.009 (4)	0.024 (5)	0.002 (4)	0.008 (4)	−0.003 (4)
C3	0.023 (4)	0.010 (4)	0.025 (5)	0.002 (3)	−0.002 (4)	0.000 (3)
C1	0.029 (4)	0.005 (4)	0.018 (5)	0.000 (3)	−0.003 (4)	−0.004 (3)
C4	0.029 (4)	0.012 (4)	0.025 (5)	−0.006 (3)	0.010 (4)	−0.002 (4)
C2	0.029 (4)	0.006 (4)	0.021 (5)	−0.001 (3)	−0.006 (3)	−0.008 (3)
Cl1	0.0314 (11)	0.0227 (13)	0.0378 (16)	−0.0032 (8)	−0.0046 (10)	−0.0067 (10)

### Geometric parameters (Å, °)

**Table:**

Pb1—O2	2.407 (7)	C6—C7	1.400 (11)
Pb1—O1	2.572 (6)	C6—C5	1.493 (12)
Pb1—O6^i^	2.586 (6)	C8—C7	1.386 (13)
Pb1—O3	2.618 (6)	C8—H8	0.9300
Pb1—O4^ii^	2.743 (6)	C7—H7	0.9300
Pb1—Cl1	2.893 (2)	C10—C11	1.376 (13)
Pb2—O5^i^	2.408 (6)	C10—H10	0.9300
Pb2—O4	2.516 (6)	C11—H11	0.9300
Pb2—O3	2.616 (6)	O5—C12	1.252 (11)
Pb2—O6^i^	2.696 (7)	O5—Pb2^vi^	2.408 (6)
Pb2—O1^iii^	2.712 (6)	O6—C12	1.248 (11)
Pb2—Cl1^iii^	3.010 (2)	O6—Pb1^vi^	2.586 (6)
O3—C5	1.250 (10)	O6—Pb2^vi^	2.696 (7)
O1—C1	1.261 (10)	C3—C4^vii^	1.381 (13)
O1—Pb2^iv^	2.712 (6)	C3—C2	1.404 (12)
O2—C1	1.276 (10)	C3—H3	0.9300
O4—C5	1.268 (9)	C1—C2	1.485 (12)
O4—Pb1^v^	2.743 (6)	C4—C2	1.393 (12)
C9—C10	1.374 (11)	C4—C3^vii^	1.381 (13)
C9—C8	1.412 (12)	C4—H4	0.9300
C9—C12	1.494 (12)	Cl1—Pb2^iv^	3.010 (2)
C6—C11	1.394 (12)		
			
O2—Pb1—O1	52.8 (2)	C10—C9—C12	120.3 (8)
O2—Pb1—O6^i^	129.8 (2)	C8—C9—C12	121.4 (7)
O1—Pb1—O6^i^	77.2 (2)	C11—C6—C7	119.8 (8)
O2—Pb1—O3	83.1 (2)	C11—C6—C5	120.5 (7)
O1—Pb1—O3	73.08 (18)	C7—C6—C5	119.6 (8)
O6^i^—Pb1—O3	77.8 (2)	C7—C8—C9	121.3 (8)
O2—Pb1—O4^ii^	86.51 (19)	C7—C8—H8	119.3
O1—Pb1—O4^ii^	136.8 (2)	C9—C8—H8	119.3
O6^i^—Pb1—O4^ii^	138.75 (19)	C8—C7—C6	119.1 (8)
O3—Pb1—O4^ii^	90.08 (19)	C8—C7—H7	120.5
O2—Pb1—Cl1	81.44 (17)	C6—C7—H7	120.5
O1—Pb1—Cl1	74.74 (15)	C9—C10—C11	122.1 (8)
O6^i^—Pb1—Cl1	90.49 (16)	C9—C10—H10	119.0
O3—Pb1—Cl1	147.46 (13)	C11—C10—H10	119.0
O4^ii^—Pb1—Cl1	117.23 (14)	O3—C5—O4	122.9 (8)
O5^i^—Pb2—O4	81.4 (2)	O3—C5—C6	118.4 (7)
O5^i^—Pb2—O3	105.8 (2)	O4—C5—C6	118.7 (7)
O4—Pb2—O3	51.03 (18)	C10—C11—C6	119.9 (8)
O5^i^—Pb2—O6^i^	50.39 (19)	C10—C11—H11	120.1
O4—Pb2—O6^i^	93.0 (2)	C6—C11—H11	120.1
O3—Pb2—O6^i^	75.9 (2)	C12—O5—Pb2^vi^	99.6 (5)
O5^i^—Pb2—O1^iii^	87.3 (2)	C12—O6—Pb1^vi^	151.2 (6)
O4—Pb2—O1^iii^	149.49 (18)	C12—O6—Pb2^vi^	86.1 (5)
O3—Pb2—O1^iii^	158.89 (17)	Pb1^vi^—O6—Pb2^vi^	99.3 (2)
O6^i^—Pb2—O1^iii^	101.5 (2)	O6—C12—O5	122.1 (9)
O5^i^—Pb2—Cl1^iii^	76.61 (15)	O6—C12—C9	119.3 (8)
O4—Pb2—Cl1^iii^	78.94 (14)	O5—C12—C9	118.5 (7)
O3—Pb2—Cl1^iii^	127.69 (14)	C4^vii^—C3—C2	120.7 (8)
O6^i^—Pb2—Cl1^iii^	126.97 (14)	C4^vii^—C3—H3	119.7
O1^iii^—Pb2—Cl1^iii^	70.88 (13)	C2—C3—H3	119.7
C5—O3—Pb1	129.5 (5)	O1—C1—O2	121.9 (8)
C5—O3—Pb2	90.4 (5)	O1—C1—C2	120.4 (7)
Pb1—O3—Pb2	100.5 (2)	O2—C1—C2	117.6 (8)
C1—O1—Pb1	89.0 (5)	C2—C4—C3^vii^	119.9 (8)
C1—O1—Pb2^iv^	122.4 (5)	C2—C4—H4	120.1
Pb1—O1—Pb2^iv^	107.7 (2)	C3^vii^—C4—H4	120.1
C1—O2—Pb1	96.2 (5)	C4—C2—C3	119.4 (8)
C5—O4—Pb2	94.6 (5)	C4—C2—C1	119.8 (8)
C5—O4—Pb1^v^	146.6 (5)	C3—C2—C1	120.7 (7)
Pb2—O4—Pb1^v^	101.2 (2)	Pb1—Cl1—Pb2^iv^	92.57 (6)
C10—C9—C8	117.9 (8)		
			
O2—Pb1—O3—C5	53.6 (7)	C11—C6—C7—C8	−2.1 (12)
O1—Pb1—O3—C5	0.4 (6)	C5—C6—C7—C8	172.8 (8)
O6^i^—Pb1—O3—C5	−79.7 (7)	C8—C9—C10—C11	−1.4 (13)
O4^ii^—Pb1—O3—C5	140.1 (7)	C12—C9—C10—C11	171.3 (8)
Cl1—Pb1—O3—C5	−8.4 (8)	Pb1—O3—C5—O4	93.5 (9)
O2—Pb1—O3—Pb2	152.9 (2)	Pb2—O3—C5—O4	−10.5 (8)
O1—Pb1—O3—Pb2	99.8 (2)	Pb1—O3—C5—C6	−87.7 (9)
O6^i^—Pb1—O3—Pb2	19.6 (2)	Pb2—O3—C5—C6	168.3 (6)
O4^ii^—Pb1—O3—Pb2	−120.60 (19)	Pb2—O4—C5—O3	11.0 (8)
Cl1—Pb1—O3—Pb2	90.9 (3)	Pb1^v^—O4—C5—O3	−107.5 (10)
O5^i^—Pb2—O3—C5	70.4 (5)	Pb2—O4—C5—C6	−167.8 (6)
O4—Pb2—O3—C5	5.7 (4)	Pb1^v^—O4—C5—C6	73.7 (12)
O6^i^—Pb2—O3—C5	111.4 (5)	C11—C6—C5—O3	166.2 (7)
O1^iii^—Pb2—O3—C5	−163.2 (5)	C7—C6—C5—O3	−8.6 (11)
Cl1^iii^—Pb2—O3—C5	−14.7 (5)	C11—C6—C5—O4	−15.0 (12)
O5^i^—Pb2—O3—Pb1	−60.0 (2)	C7—C6—C5—O4	170.3 (7)
O4—Pb2—O3—Pb1	−124.7 (3)	C9—C10—C11—C6	0.0 (13)
O6^i^—Pb2—O3—Pb1	−18.97 (19)	C7—C6—C11—C10	1.8 (13)
O1^iii^—Pb2—O3—Pb1	66.4 (6)	C5—C6—C11—C10	−173.0 (8)
Cl1^iii^—Pb2—O3—Pb1	−145.12 (11)	Pb1^vi^—O6—C12—O5	88.8 (13)
O2—Pb1—O1—C1	−2.0 (5)	Pb2^vi^—O6—C12—O5	−13.2 (9)
O6^i^—Pb1—O1—C1	173.1 (5)	Pb1^vi^—O6—C12—C9	−94.5 (15)
O3—Pb1—O1—C1	92.2 (5)	Pb2^vi^—O6—C12—C9	163.4 (7)
O4^ii^—Pb1—O1—C1	20.9 (6)	Pb2^vi^—O5—C12—O6	15.0 (10)
Cl1—Pb1—O1—C1	−92.8 (5)	Pb2^vi^—O5—C12—C9	−161.6 (6)
O2—Pb1—O1—Pb2^iv^	121.8 (3)	C10—C9—C12—O6	−167.9 (8)
O6^i^—Pb1—O1—Pb2^iv^	−63.0 (2)	C8—C9—C12—O6	4.6 (13)
O3—Pb1—O1—Pb2^iv^	−144.0 (2)	C10—C9—C12—O5	8.9 (13)
O4^ii^—Pb1—O1—Pb2^iv^	144.8 (2)	C8—C9—C12—O5	−178.7 (8)
Cl1—Pb1—O1—Pb2^iv^	31.07 (15)	Pb1—O1—C1—O2	3.6 (8)
O1—Pb1—O2—C1	2.0 (4)	Pb2^iv^—O1—C1—O2	−106.9 (8)
O6^i^—Pb1—O2—C1	−4.1 (6)	Pb1—O1—C1—C2	−174.6 (7)
O3—Pb1—O2—C1	−72.0 (5)	Pb2^iv^—O1—C1—C2	75.0 (9)
O4^ii^—Pb1—O2—C1	−162.5 (5)	Pb1—O2—C1—O1	−3.9 (9)
Cl1—Pb1—O2—C1	79.3 (5)	Pb1—O2—C1—C2	174.4 (6)
O5^i^—Pb2—O4—C5	−124.1 (5)	C3^vii^—C4—C2—C3	0.8 (14)
O3—Pb2—O4—C5	−5.6 (4)	C3^vii^—C4—C2—C1	−179.8 (7)
O6^i^—Pb2—O4—C5	−74.9 (5)	C4^vii^—C3—C2—C4	−0.8 (14)
O1^iii^—Pb2—O4—C5	166.5 (4)	C4^vii^—C3—C2—C1	179.8 (8)
Cl1^iii^—Pb2—O4—C5	158.0 (5)	O1—C1—C2—C4	0.5 (12)
O5^i^—Pb2—O4—Pb1^v^	26.4 (2)	O2—C1—C2—C4	−177.7 (7)
O3—Pb2—O4—Pb1^v^	144.8 (3)	O1—C1—C2—C3	179.9 (7)
O6^i^—Pb2—O4—Pb1^v^	75.6 (2)	O2—C1—C2—C3	1.7 (12)
O1^iii^—Pb2—O4—Pb1^v^	−43.1 (5)	O2—Pb1—Cl1—Pb2^iv^	−79.96 (15)
Cl1^iii^—Pb2—O4—Pb1^v^	−51.54 (15)	O1—Pb1—Cl1—Pb2^iv^	−26.31 (15)
C10—C9—C8—C7	1.1 (12)	O6^i^—Pb1—Cl1—Pb2^iv^	50.27 (17)
C12—C9—C8—C7	−171.5 (8)	O3—Pb1—Cl1—Pb2^iv^	−17.5 (3)
C9—C8—C7—C6	0.6 (12)	O4^ii^—Pb1—Cl1—Pb2^iv^	−161.53 (15)

Symmetry codes: (i) *x*, −*y*+3/2, *z*−1/2; (ii) *x*−1, *y*, *z*; (iii) −*x*+1, *y*+1/2, −*z*+1/2; (iv) −*x*+1, *y*−1/2, −*z*+1/2; (v) *x*+1, *y*, *z*; (vi) *x*, −*y*+3/2, *z*+1/2; (vii) −*x*+1, −*y*+1, −*z*+1.
